# Local distribution analysis of cytotoxic molecules in liver allograft is helpful for the diagnosis of acute cellular rejection after orthotopic liver transplantation

**DOI:** 10.1186/1746-1596-7-148

**Published:** 2012-10-30

**Authors:** Long Cheng, Fuzhou Tian, Lijun Tang, Shuguang Wang, Geng Chen, Guangjie Duan, Xiaochu Yan

**Affiliations:** 1 Institute of Pathology, Southwest Hospital, Third Military Medical University, Chongqing, 400038, China; 2 Department of General Surgery, General Hospital of Chengdu Military Command, Chengdu, 610083, China; 3 Institute of Hepatobiliary Surgery, Southwest Hospital, Third Military Medical University, Chongqing, 400038, China

**Keywords:** Liver transplantation, Acute cellular rejection (ACR), Rejection activity index (RAI), Perforin, Granzyme B, T-cell intracellular antigen-1

## Abstract

**Background:**

As it is often difficult for a transplant pathologist to make a definite diagnosis of acute cellular rejection (ACR) by routine morphological analysis of liver allograft biopsy, supplementary methods and objective markers are needed to facilitate this determination.

**Methods:**

To evaluate the diagnostic value of cytotoxic molecules in ACR episodes, immunohistochemical staining for perforin, granzyme B and T-cell intracellular antigen-1 (TIA-1) were performed in liver allograft biopsies. The positive cells in the portal tract area and lobules were counted separately to investigate the distribution of the cytotoxic molecules.

**Results:**

The immunohistochemical study showed that the overall positive rates for the three markers were not significantly different between the ACR and non-ACR groups. However, in the portal tract area, perforin-, granzyme B- and TIA-1-positive cells in the ACR group were significantly more than those in the non-ACR groups. In the lobules, perforin- and granzyme B-positive cells in the ACR group were significantly more than those in the biliary complication and opportunistic infection groups, while TIA-1-positive cells was significantly fewer than those in non-ACR groups. The numbers of positive cells in the portal tract area correlated with the rejection activity index of ACR.

**Conclusions:**

These results indicate that, though the overall positive rates have nonsense in ACR diagnosis, the quantification and local distribution analysis of cytotoxic molecule positive cells in liver tissue is helpful for differential diagnosis and severity evaluation of ACR following liver transplantation.

**Virtual slides:**

The virtual slide(s) for this article can be found here: http://www.diagnosticpathology.diagnomx.eu/vs/2292255038100487

## Introduction

With the incidence reportedly ranging from 30% to 70%, acute cellular rejection (ACR) is one of the most common complications after orthotopic liver transplantation (OLT) [1,2]. The appropriate immunosuppressive therapy for ACR, which is important for reducing morbidity and improving the life quality of recipients, is based on precise diagnoses and grading. At present, the Banff schema is accepted as the diagnostic judge standard for ACR, which is morphologically characterized by lymphocyte infiltration of portal tracts, bile duct damage and endothelitis in portal and hepatic central veins [3-5]. However, due to the overlapping histological features between ACR and other complications following liver transplantation, differential diagnoses and severity evaluations for ACR are often difficult. This prompted us to look for some potential methods and molecular markers helpful for diagnosing ACR and evaluating its severity. 

**Table 1:** Underlying diseases of the transplant recipients

**Primary disease**	**No. of patients**
Posthepatitic cirrhosis	17
Hepatocellular carcinoma	15
Posthepatitic cirrhosis complicated by hepatocellular carcinoma	18
Severe hepatitis	17
Alcoholic cirrhosis	2
Primary biliary cirrhosis	2
Primary hepatic amyloidosis	2

It is generally accepted that T cell-mediated immune reactions play a pivotal role in the pathogenesis of ACR, and CD8+ cytotoxic T cells induce target cell death during acute allograft rejection in liver allograft tissues [6-8]. Cytotoxic molecules such as perforin, granzyme B and T-cell intracellular antigen-1 (TIA-1) are present in the cytoplasmic granules of cytotoxic T cells and function at the effector end of the acute rejection process [9]. Nevertheless, a study also showed that cytotoxic molecules can also mediate liver graft rejection in the absence of CD8+ T cells [10]. Thus, cytotoxic protein detection might be a sensitive and objective method for predicting acute rejection injury. It has been reported that granzyme B and perforin played predictive roles in acute rejection diagnosis after renal, heart and intestinal transplantation [11-14]. Moreover, in acute rejection after kidney transplantation, the quantity and intensity of TIA-1 expression are both increased, and this variation can reflect rejection severity to some extent [15]. However, the diagnostic value of these cytotoxic molecules in acute cellular rejection after liver transplantation has not yet been clearly elucidated.

To further evaluate the role of cytotoxic molecules in ACR diagnosis, immunohistological staining of perforin, granzyme B and TIA-1 was performed on allograft liver biopsies. As it was noted that different liver diseases mainly target at different tissues and cells of liver, the positive cells in the portal tract area and lobules were counted separately to investigate the local distribution characteristics of the cytotoxic molecules. Meanwhile, correlations between the numbers of positive cells and the Banff rejection activity index (RAI) were analyzed.

## Materials and methods

### Patients and clinic materials

The liver tissue samples were obtained from the Institute of Hepatobiliary Surgery of Southwest Hospital, the Third Military Medical University. Written informed consent was obtained from all patients and this study was carried out in accordance with the principles of the Helsinki Declaration and approved by the Ethical Committee of the Third Military Medical University, Chongqing, Peoples Republic of China.

Between February 2000 and December 2006, 234 samples were obtained by percutaneous needle biopsy from patients that underwent orthotopic liver transplantation (OLT) in Southwest hospital. A total of 108 biopsy samples from 73 patients (66 males and 7 females) were enrolled in the stud**y** based on the following criteria: those with more than 10 portal tracts in each biopsy; those with integrated clinical follow-up information. The ages of the patients ranged from 18 to 69 years and their primary clinical diagnoses are listed in Table 1.

All patients enrolled in the study were treated with glucocorticoid, Mycophenolate Mofetil and cyclosporine A or tacrolimus after surgery. Glucocorticoid was stopped within three months, and cyclosporine A or tacrolimus was administered separately to maintain immunosuppression. Complications were diagnosed based on clinical and biochemical data in combination with pathological evaluation of allograft liver needle biopsy specimens. ACR episodes were generally treated with daily administration of methylprednisolone (20mg/kg) for three consecutive days.

### Histological observation

Biopsy specimens were fixed in 10% buffered formalin, embedded in paraffin, sectioned serially at a 4μm thickness, and de-waxed. H&E was performed routinely. The RAI for each specimen was scored according to the Banff consensus by two independent qualified transplant pathologists unaware of the clinical data of the patients [3].

### Immunohistological staining

Monoclonal antibodies against perforin, granzyme B (DAKO, Glostrup, Denmark) and TIA-1 (abcam, Cambridge, UK.) were applied to the above sections. Antigens were retrieved in citrate buffer in a microwave oven and endogenous peroxidase activity was blocked with 3% hydrogen peroxide. Then, sections were incubated at 4°C with primary antibodies (with a dilution of 1:200 for perforin, 1:200 for granzyme B and 1:100 for TIA-1) overnight and the Envision™ staining (DAKO, Glostrup, Denmark) procedure was performed. Sections with the primary antibody application omitted served as a negative control.

Brown granular staining was considered to be a positive signal for the IHC assay. The positive cells were counted under light microscope according to the previous description with some modifications [8]. The positive cells in the portal tract area and lobules were counted separately to investigate the distribution of the cytotoxic molecules. For each slide, the positive cells in least 10 portal tracts and 10 high power fields (HPF) in the lobules were counted under the light microscope (Olympus BX51, Japan). The average positive cells per portal tract and per HPF in the lobules were calculated.

### Statistical analysis

Statistical analyses were performed using SPSS Version 13.0 for Windows (Statistical Package for the Social Sciences; SPSS, Munich, Germany). Groups were compared using Mann-Whitney *U*-tests, and Spearman’s Rank test was performed to look for correlations between the number of positive cells and RAI score. *P*<0.05 was considered statistically significant.

## Results

### Histological observation

**Figure 1:**
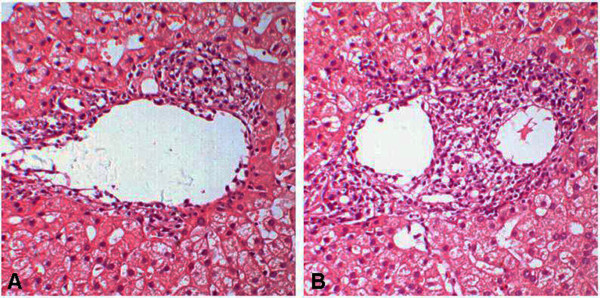
**The histopathological features of acute cellular rejection.** Typical histopathological changes, including portal inflammation, bile duct inflammation/damage and venous endotheliatis, so called “triads,” appeared in mild (**A**, RAI = 4) and moderate (**B**, RAI = 7) acute cellular rejection (H&E, ×200).

In the 108 cases, 62 cases were diagnosed as ACR and 46 were non-ACR, including 19 biliary complications (BC), 6 ischemic/reperfusion injuries (I/R), 6 opportunistic infections (OI) and 15 undefined complications (UD). In the ACR cases, varying degrees of lymphocytic infiltration of portal tracts, bile duct damage and subendothelialitis were recognized in the pathologically examined specimens. The numbers of samples with RAI scores of 1, 2, 3, 4, 5, 6, 7 and 8 were 4, 9, 9, 15, 9, 6, 6 and 4 cases respectively. (Figure 1A-B).

### The expression and distribution of cytotoxic effector molecules in ACR and non-ACR tissue

**Figure 2:**
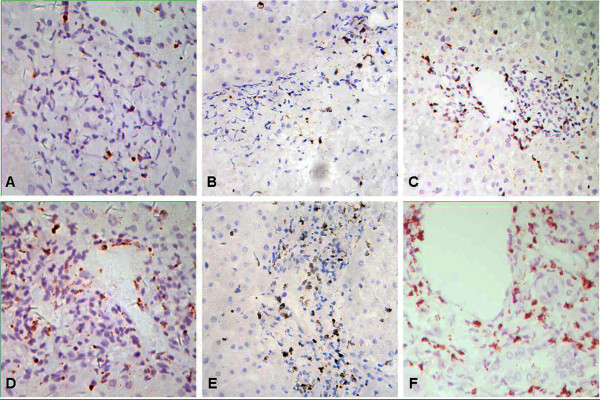
**Immunostaining features of perforin, granzyme B and TIA-1 in the portal tract area.** The expression of perforin (**A**), granzyme B (**B**) and TIA-1 (**C**) in the portal tract area in biopsies without ACR is sporadic and significantly less than that with ACR (**D**, **E** and **F**, respectively). (Immunostaining with hematoxylin counter staining, ×200).

**Figure 3:**
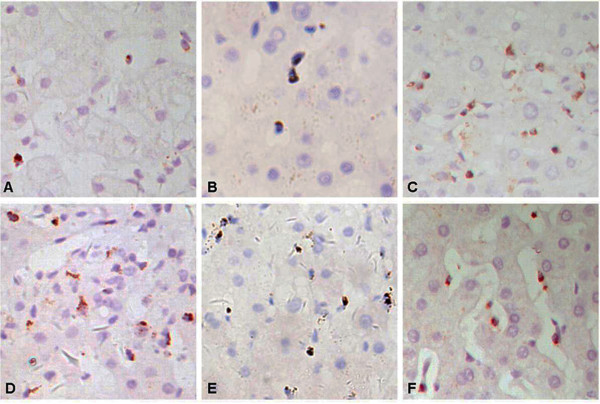
**Immunostaining features of perforin, granzyme B and TIA-1 in lobules.** The expression of perforin (**A**) and granzyme B (**B**) in lobules of biopsies without ACR is sporadic and significantly less than that with ACR (**D** and **E**, respectively). However, the expression of TIA-1 in lobules of biopsies without ACR **(C)** is prevalent, more than that with ACR **(F)**. (Immunostaining with hematoxylin counter staining, ×400).

**Figure 4:**
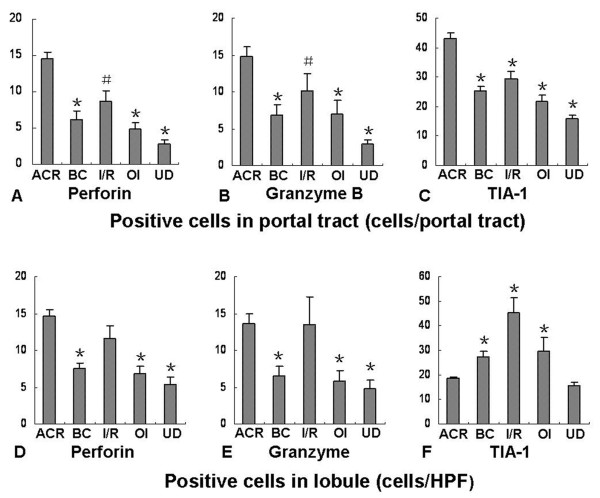
**The numbers of perforin-, granzyme B- and TIA-1-positive cells in liver biopsies after liver transplantation.** The numbers of perforin- and granzyme B-positive cells in the portal tract area in the ACR group were greater than in the non-ACR groups (**A**, **B**), while in lobules there were more positive cells in the ACR group than in the BC, OI and UD groups, but a similar amount to that in the I/R group (**D**, **E**). There were significantly more TIA-1-positive cells in the portal tract area in the ACR group than in the non-ACR groups (**C**), but in lobules there were significantly fewer in the ACR group than in the BC, I/R or OI groups (**F**). ACR, acute cellular rejection; BC, biliary complications; I/R, ischemic/reperfusion injury; OI, opportunistic infection; UD, undefined complications. ^*^P<0.01, ^#^P<0.05, compared with ACR.

Perforin, granzyme B and TIA-1 were mainly located in the cytoplasm of inflammatory cells in portal tracts and lobules, and they were frequently observed in the epithelia of interlobular bile ducts and subendothelial portions of portal veins in ACR, (Figure 2 and 3). There was no significant difference in the positive rates for perforin, granzyme B and TIA-1 between ACR and non-ACR samples (98.4%% versus 95.6%, 96.8% versus 91.3% and 100% versus 100%, respectively). However, the number and distribution of positive cells were different. There were significantly more perforin-, granzyme B- and TIA-1-positive cells in the portal tract area in the ACR group than in the non-ACR groups, and there was no significant difference among the non-ACR groups. The numbers of perforin- and granzyme B-positive cells in lobules in the ACR group were significantly greater than those in the BC, OI and UD groups, but they were similar to that in the I/R group. The numbers of TIA-1-positive cells in lobules in the ACR group was significantly lower than those in BC, I/R and OI groups, but was similar to that in UD group. (Figure 4) The above results indicate that the positive cell number, but not the positive rate of cytotoxic molecules in liver biopsies, could be used as a statistical indicator of ACR.

### Expression of cytotoxic effector molecules correlates with RAI

**Figure 5:**
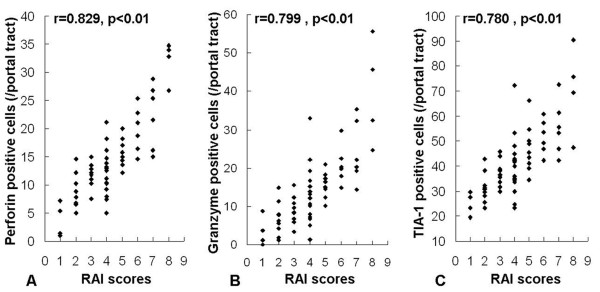
**Correlations between perforin-, granzyme B- and TIA-1-positive cells in the portal tract area and the RAI scores.** Values for each sample are plotted in each column (**A**, **B** and **C**, respectively).

In the ACR group, we recognized a tendency for the number of positive cells to increase as the RAI score rose, so the relationship between the number of positive cells and RAI score was further analyzed. There were a significant positive correlations between the numbers of positive cells in portal tracts and the RAI scores (r=0.829 for perforin, 0.799 for granzyme B and 0.780 for TIA-1, respectively), (Figure 5). The numbers of positive cells in lobules were not correlated with the RAI score (p>0.05). These results indicate that immunohistochemical analysis of cytotoxic molecules in the portal tract area of liver might be a useful supplementary tool for the objective evaluation of ACR severity.

## Discussion

Acute cellular rejection (ACR) is still common after liver transplantation (OLT) despite well developed immunosuppressive agents, with incidence ranging from 30% to 70% in different series. Multivariate analysis showed that recipient age, underlying liver disease, and Child’s class before LT were independently associated with the development of ACR [16]. Although the pathogenesis of ACR remains need to be further elucidated, it is generally accepted that ACR occurrence is mainly due to recognition of donor alloantigen by recipient T lymphocytes. Following recognition and activation, T lymphocytes trigger a series of immunoresponses and effect mechanisms. In most cases, ACR responds well to immunosuppressive treatment. However, this should not lead to an underestimation of its importance because immunosuppressive treatment is associated with increased risk for infections, recurrence of virus hepatitis, and metabolic complications such as diabetes mellitus, hyperlipidemia, and hypertension, etc. On the other hand, the repeated ACR episodes without immunosuppressive treatment or with inadequate immunosuppressive therapy might induce the occurrence of chronic rejection which would result in graft loss. So appropriate immunosuppressive therapy for ACR, is critical for reducing morbidity and improving the life quality of recipients, and makes it necessary to diagnose ACR timely and definitely.

Berman et al. summarized the histopathologic features of ACR in three aspects which constitute the basis of Banff schema: 1) mixed infiltration of inflammatory cells, including mainly mononuclear cells and also various amounts of neutrophils and eosinophils, in the portal area; 2) endothelialitis in portal and hepatic central veins characterized by subendothelial infiltration of inflammatory cells; and 3) bile duct damage with cholangitis and degenerative necrosis of biliary epithelial cells. At present, allograft biopsy remains the ‘gold standard’ for diagnosing ACR, and the Banff schema is accepted by pathologists as the diagnosing and grading criterion for ACR. However, there are overlapping histological features and clinical manifestation between ACR and other complications following liver transplantation [17], such as ischemic/reperfusion injury, biliary complication, recurrent virus hepatitis, etc. These overlaps make ACR diagnosis and grading often difficult, and urge us to explore some potential methods and molecular markers helpful for diagnosing ACR and evaluating its severity.

Perforin and granzyme B are proteins in the cytoplasmic granules of T lymphocytes and natural killer cells. Upon release by exocytosis, perforin disrupts lipid membranes and granzyme B accesses the cytosol of target cells, subsequently triggering cell death through apoptosis [18,19]. Animal experiments and clinical studies found that perforin and granzyme B were overexpressed after liver transplantation, which suggested a role in pathogenesis of acute rejection [20,21]. Studies have reported that perforin and granzyme B can be sensitive and specific markers for diagnosing ACR [22,23]. Inconsistent with these reports, in our cohort, perforin and granzyme B were expressed widely in liver allograft biopsies with or without ACR, and no difference in expression rate was observed. This inconsistence may arise from differences in the examination methods and judging criteria. Because of the non-utility of the positive rate for ACR diagnosis, in current study, we focused on quantifying the positive cells and determining their distribution in liver tissue. Our results showed that the numbers of perforin- and granzyme B-positive cells in the portal tract area were significantly greater with ACR than with other complications. However, in the lobule area, there were more positive cells with ACR than with BC or OP, but a similar amount of cells to that with I/R injury. These results are consistent with reports stating that ACR involves not only damage to the portal tract but also lobule injury, and that the cytotoxic T cells are also activated during I/R injury [24,25]. Our study indicates that the quantification of perforin- and granzyme B-positive cells in different areas in liver biopsies could be more informative for ACR diagnosis than mere determination of the positive rate.

Unlike perforin and granzyme B, which are expressed in cytotoxic T cells and NK cells, TIA-1, also named granule membrane protein-17 (GMP-17), is expressed not only by cytotoxic T cells and NK cells but also by monocytes and neutrophils [26,27]. TIA-1 is the granule component responsible for inducing DNA fragmentation and apoptosis in cytolytic lymphocyte targets. In acute rejection after kidney transplantation, the quantity and intensity of TIA-1 expression increase, and the degree of this variation can reflect rejection severity to some extent [15,28,29]. However, the diagnostic value of TIA-1 in acute rejection after liver transplantation has not been determined. Our data show that TIA-1 is also expressed widely in liver allograft biopsies with or without ACR. Interestingly, our results showed that the number of TIA-1-positive cells significantly increased in the portal tract area but not in lobules during ACR episodes. By contrast, during other complications such as biliary complications, opportunistic infections, and preservation/reperfusion injuries, TIA-1-positive cells significantly increased in lobules but not in the portal tract area. These opposite alterations may occur because cytotoxic T cells are prevalent in the portal tract in ACR cases, while monocytes and neutrophils, which also release TIA-1, are more popular in lobular in biliary complications, opportunistic infections, and preservation/reperfusion injuries. This distributional difference of TIA-1-positive cells implies that a marked increase in the portal tract combined with insignificant changes in lobules may indicate a diagnosis of ACR. To our knowledge, no previous studies found this pattern of TIA-1 expression in liver grafts. Therefore, the local distribution of TIA-1-positive cells in liver biopsies may provide a morphological means of distinguishing ACR from other complications after liver transplantation.

One study showed that the number of CD8-positive cells correlated with rejection severity in liver allograft tissues.^8^ However, the relationship between rejection severity and the expression of perforin, granzyme B and TIA-1 has not been determined. Our present data show that the numbers of perforin-, granzyme B- and TIA-1-positive cells in the portal tract area correlated with acute rejection severity after liver transplantation. This result is compatible with the notion that the portal tracts are the main targets of ACR and that these cytotoxic molecules are at the effector end of the acute rejection process. Based on our results, we conclude that the identification of perforin, granzyme B and TIA-1 in the portal tract area of liver biopsies would be helpful for determining the severity of ACR.

Immunohistochemical staining has become a routine method in clinical pathological diagnosis. Immunohistochemical assays for perforin, granzyme B and TIA-1 are applicable in most pathology laboratories of large hospitals. ACR pathological diagnoses are based on H&E findings, according to the Banff schema. Our result raise the possibility that immunohistochemical analysis of cytotoxic molecules has the potential to become a supplementary as well as an objective assessment method for ACR diagnosis to be used as an adjunct to the Banff schema in the future.

In conclusion, our results indicate that, though the overall positive rates have nonsense in ACR diagnosis, the quantification and local distribution analysis of cytotoxic molecule positive cells in liver tissue is helpful for differential diagnosis and severity evaluation of ACR following liver transplantation.

## Abbreviations

ACR: Acute cellular rejection; RAI: Rejection activity index; TIA-1: T-cell intracellular antigen-1; BC: Biliary complications; IRI: Ischemic/reperfusion injuries; OI: Opportunistic infections; UD: Undefined complications.

## Competing interests

The authors declare that they have no competing interests.

## Authors’ contributions

LC, FT and LT contributed to the research designing, writing of the paper, performing of the research and data analysis. SW, GC and GD participated in the performance of the research. XY participated in research design and the writing of the paper. All authors read and approved the final manuscript.
